# Traction-assisted endoscopic submucosal dissection for a terminal ileal neuroendocrine tumor

**DOI:** 10.1055/a-2913-8649

**Published:** 2026-07-29

**Authors:** Lu Wang, Lin Liu, Feilong Tian, Meidong Xu, Dongwu Jia, Mizhu Wang, Zhenyu Jiang

**Affiliations:** 1 Department of GastroenterologyThe Second Affiliated Hospital of Baotou Medical CollegeBaotouInner MongoliaChina; 2 Endoscopy Center, Department of GastroenterologyShanghai East Hospital, Tongji University School of MedicineShanghaiChina

Neuroendocrine tumors (NETs) are relatively uncommon but clinically significant neoplasms originating from neuroendocrine cells distributed throughout the gastrointestinal tract. 1​ ​2​ Small-bowel NETs, particularly those arising in the terminal ileum, often manifest as small, inconspicuous subepithelial lesions that are easily overlooked during routine endoscopy due to their subtle appearance and intact overlying mucosa. 3​ ​4​ Endoscopic submucosal dissection (ESD) has emerged as an effective and minimally invasive technique for the en bloc resection of gastrointestinal submucosal tumors, providing reliable histopathological assessment with accurate margin evaluation while preserving organ integrity and avoiding the morbidity associated with surgical resection. 5​

**Fig. 1:**
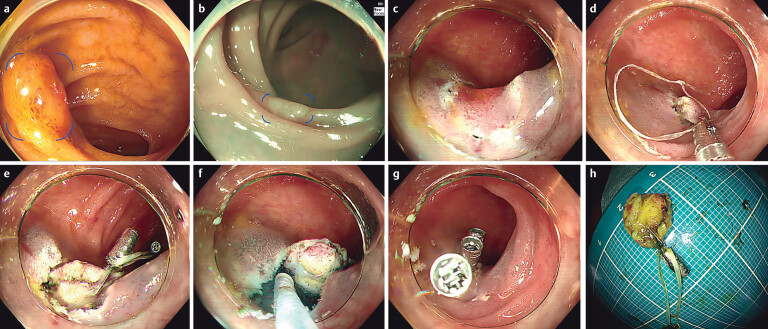
Initial endoscopic findings and a traction-assisted ESD procedure. ( **a** ) Initial colonoscopy showing a protruded erosive lesion in the terminal ileum. ( **b** ) Two small sigmoid polypoid lesions with a NICE type 2 pattern, removed with biopsy forceps. ( **c** ) Submucosal injection and circumferential mucosal incision around the lesion. ( **d** – **f** ) Two-point sustained traction created using endoclips and a plastic loop, providing adequate exposure of the submucosal dissection plane. ( **g** ) Endoclip closure of the post-ESD mucosal defect. (h) An en bloc resected specimen.

**Fig. 2:**
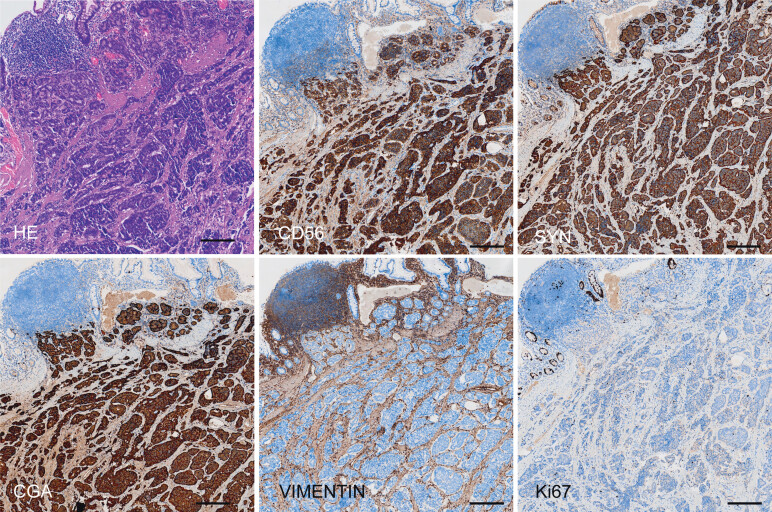
Histopathological and immunohistochemical findings. ( **a** ) Histopathology of the resected terminal ileal lesion, consistent with a neuroendocrine neoplasm and favoring NET G1 on integrated evaluation. ( **b** ) Ki-67 labeling index of approximately 1%. ( **c** ) Negative staining for Vimentin. ( **d** ) Positive staining for synaptophysin. ( **e** ) Positive staining for chromogranin A. ( **f** ) Positive staining for CD56.

In this case, a 36-year-old man underwent colonoscopy, and a small subepithelial lesion was incidentally identified in the terminal ileum, endoscopically suspected to be a neuroendocrine tumor. Given the small lesion size and absence of any evidence of lymph node or distant metastasis on preoperative evaluation, traction-assisted ESD was selected as the treatment strategy to achieve en bloc resection for precise pathological staging while avoiding unnecessary surgical intervention. Conventional ESD in the narrow lumen of the terminal ileum is technically challenging owing to poor visualization, limited working space, thin bowel wall, and unstable endoscope position. To overcome these difficulties, we employed a simple traction method using endoclips and a plastic loop, which provided sustained countertraction, significantly improved the exposure of the submucosal dissection plane, and facilitated safe, controlled dissection. No intraprocedural or postprocedural adverse events, such as bleeding, perforation, or abdominal pain, occurred ( Fig. 1 ). Histopathological examination with immunohistochemistry confirmed a grade 1 neuroendocrine tumor (NET G1) with negative resection margins ( Fig. 2 ; Video 1​ ). This case illustrates that traction-assisted ESD is a feasible, safe, and minimally invasive therapeutic option for carefully selected patients with small terminal ileal NETs, particularly when surgery may be considered overtreatment.

**Video 1** Traction-assisted endoscopic submucosal dissection of a small terminal ileal neuroendocrine tumor, demonstrating loop-and-clip traction, submucosal dissection, en bloc resection, and histopathological confirmation.


Endoscopy_UCTN_Code_TTT_1AQ_2AD_3AD
